# Controlling the Nucleation and Growth of Salt from Bodily Fluid for Enhanced Biosensing Applications

**DOI:** 10.3390/bios13121016

**Published:** 2023-12-06

**Authors:** Siddharth Srivastava, Yusuke Terai, Jun Liu, Giovanni Capellini, Ya-Hong Xie

**Affiliations:** 1 Department of Materials Science and Engineering, University of California, Los Angeles, CA 90095, USA; 2 Department of Micro-Nano Mechanical Science and Engineering, Nagoya University, Nagoya 464-8601, Japan; 3 IHP—Leibniz Institute for High Performance Microelectronics, 15236 Frankfurt (Oder), Germany; giovanni.capellini@uniroma3.it; 4 Department of Science, Università Degli Studi Roma Tre, Viale Marconi 446, 00146 Rome, Italy; 5 Jonsson Comprehensive Cancer Center, University of California, Los Angeles, CA 90095, USA

**Keywords:** SERS, biosensing, extracellular vesicles, crystallization, plasmonics, nucleation

## Abstract

Surface-enhanced Raman spectroscopy (SERS) represents a transformative tool in medical diagnostics, particularly for the early detection of key biomarkers such as small extracellular vesicles (sEVs). Its unparalleled sensitivity and compatibility with intricate biological samples make it an ideal candidate for revolutionizing noninvasive diagnostic methods. However, a significant challenge that mars its efficacy is the throughput limitation, primarily anchored in the prerequisite of hotspot and sEV colocalization within a minuscule range. This paper delves deep into this issue, introducing a never-attempted-before approach which harnesses the principles of crystallization—nucleation and growth. By synergistically coupling lasers with plasmonic resonances, we navigate the challenges associated with the analyte droplet drying method and the notorious coffee ring effect. Our method, rooted in a profound understanding of crystallization’s materials science, exhibits the potential to significantly increase the areal density of accessible plasmonic hotspots and efficiently guide exosomes to defined regions. In doing so, we not only overcome the throughput challenge but also promise a paradigm shift in the arena of minimally invasive biosensing, ushering in advanced diagnostic capabilities for life-threatening diseases.

## 1. Introduction

Numerous life-threatening diseases demand the advancement of efficient, reliable, and noninvasive methods for early diagnosis and the precise identification of key biomarkers such as those embodied within small extracellular vesicles (sEVs) [1,2,3]. Surface-enhanced Raman spectroscopy (SERS) has emerged as a powerful technique for biosensing applications, leveraging the plasmonic resonance of metallic nanostructures such as gold and silver. By employing these plasmonic materials, SERS boosts the inherently weak Raman signals by several orders of magnitude through localized surface plasmon resonance (LSPR), offering at once high molecular specificity and high sensitivity [4,5]. The plasmonic enhancement, resulting from the collective oscillation of free electrons at the metal–dielectric interface, enables the detection of vibrational information from biological substances with extraordinary precision. Along with localized electromagnetic-field enhancement and nanostructure-geometry tuning, this plasmonic effect has rendered SERS invaluable for disease detection, including the differentiation of vesicles from diverse cellular origins, and positions it as a pivotal tool for sEV biomarker identification [3,4]. Indeed, SERS features extraordinary sensitivity and specificity, label-free detection, rich information content, single-vesicle detection capability, real-time analysis, compatibility with complex biological samples, broad applicability across various diseases, and potential for integration and automation [3,4].

Presently, the monumental challenges [6] in EV-based liquid biopsy, which holds significant promise for revolutionizing diagnostics [7,8,9], are addressed by harnessing the unmatched strengths of SERS [6,10,11]. The sEV-based liquid biopsy technique, with its adept ability, uniquely addresses the inherent obstacles, particularly low throughput, paving the way for accelerated and enhanced medical diagnostics. Utilizing SERS for this purpose could enable a world where a routine blood test to catch individual cancer-derived exosomes, differentiate exosome subtypes for enhanced therapeutic efficacy, or determine virus variants could be possible. Such a reality would not only be groundbreaking but transformative for healthcare across the globe. In this realm, SERS plays a pivotal role. However, the challenges are immense, with the throughput limitation standing tall among them. One might question, how does our technique assist liquid biopsy in navigating this intricate landscape? The answer lies in making two mammoth strides forward: firstly, by significantly increasing the areal density of the available plasmonic hotspots (i.e., not covered by precipitates), and secondly, by congregating those exosomes, herding them effectively to a defined region. This paper embarks on this very journey, driven by an in-depth grasp of the materials science of crystallization; it delves into the fundamental principles governing the kinetics of nucleation, growth, and the organization of atoms or molecules into crystalline structures and how these principles can be harnessed to optimize the processes and applications in our study.

One of the paramount challenges facing SERS, particularly when used to analyze EVs in a label-free fashion, is the throughput limitation [4,12]. Throughput in this context refers to the statistically significant number of high-quality or high signal-to-noise spectra that can be obtained from the biosample within a given timeframe. The key limiting factor in achieving high throughput and ensuring the reliability and relevance of the analysis is the colocalization of SERS hotspots with sEVs under test at the sub-µm scale, once the sample has been deposited on the SERS substrate by, for example, the droplet drying method.

In biosensing, the popular analyte droplet drying method refers to the deposition of small volumes of liquid containing the biological sample of interest onto a substrate analogous to the methods commonly employed in medical laboratories [13,14,15]. The evaporation of the droplets leads to the formation of distinct patterns due to the crystallization process of the material in solution/suspension in the dropped fluid, which leads inevitably to the biological entities of interest (e.g., sEVs) to aggregate to specific regions of the pattern. Recently, the droplet drying method has seen growing popularity owing to its reproducibility and ease of integration with various detection platforms [14]. Furthermore, it enables enhanced control over the analyte concentration, facilitated handling, and it allows to analyze minute quantities of biological substances [15,16].

In this paper, we investigate the challenges due to the colocalization in SERS, and we propose a never-attempted-before approach leveraging two fundamental aspects of the crystallization occurring in a drying droplet—nucleation and growth—to improve the throughput of the SERS technique. We shall demonstrate how the local amplification of the laser electromagnetic-field intensities due to the SERS substrate can be leveraged to favor the nucleation and growth of the analyte to induce spatially controlled precipitation. This groundbreaking approach provides a potential avenue to process a greater number of biosamples with micrometer precision, setting the stage for an enhanced analytical efficiency.

Central to our research is the coffee ring effect [17,18,19], a phenomenon observed during the drying of droplets, characterized by an interplay of diffusion, convection, deposition, and molecule–substrate surface interaction. This intricate and yet to be fully understood process results in the deposition of particles at the droplet’s edge, creating a ring-like pattern, a consequence of complex interactions such as capillary action and Marangoni flow [20]. The situation becomes even more convoluted when considering bioanalytes, where different types of particles—some forming solutions, others suspensions, and still others colloidal—each exhibit unique dynamics. The variability and interplay among these components create a highly complex environment within the droplet. This complexity plays a crucial role in the local concentration of analytes and the formation of “hotspots” for SERS detection. In our study, we embrace and explore this complexity, seeking ways to control and leverage the coffee ring effect to enhance biosensing applications.

By merging the promising field of SERS with the controlled and well-understood crystallization process, our research endeavors to contribute a novel and sophisticated method to the growing landscape of noninvasive biosensing technologies. The innovative utilization of the coffee ring effect and the analyte droplet drying method represents an interdisciplinary approach that transcends the existing challenges. The forthcoming sections will delve into the theoretical underpinnings, experimental design, results, and implications of our findings in controlling the nucleation and growth of salt from bodily fluid for enhanced biosensing applications. The potential impact is vast, signaling a paradigm shift in the early detection and diagnosis of life-threatening diseases.

## 2. Materials and Methods

### 2.1. SERS Substrate Fabrication

The SERS platform used in this study was previously developed, with the schematic and characterization results provided in our prior work [20,21,22,23]. The fabrication process is as follows:

- Formation of Polystyrene Ball Layer: A single layer of self-assembled polystyrene (PS) balls (500 nm) was generated using Langmuir–Blodgett patterning.
- Transfer and Deposition: The layer was transferred to a 4″ (001) silicon wafer with 50 nm SiO_2_ deposited on top, followed by a deposition of 50 nm Cr.
- PS Ball Removal: PS balls were removed using chloroform, and the SiO_2_ was exposed using reactive-ion etching.
- Silicon Etching: The silicon was etched using KOH to create inverted nanopyramids with 57.5°-angle sidewalls, exploiting the different etching rates along the [001] and [111] silicon directions.
- Gold Film Deposition: A 200 nm film of gold was deposited on the pitted surface using electron beam deposition and bonded to a carrier wafer using epoxy before lifting off.

### 2.2. sEV Isolation Procedure

The sEV isolation procedure was performed as follows:

- Preliminary Centrifugation: Cell culture supernatants were first centrifuged at 300 g (4 °C, 10 min) and 2000 g (4 °C, 15 min) to remove cells and apoptotic bodies.
- Further Centrifugation: Supernatants were centrifuged at 12,000 g (4 °C, 45 min) to remove cell debris, followed by filtration using 0.22 μm-pore filters.
- Ultracentrifugation: Supernatants were ultracentrifuged at 110,000 g (4 °C, 70 min), and the pellets were resuspended in prechilled PBS. The process was repeated, and the final pellets were resuspended in 50–100 μL of PBS for NTA measurement.

### 2.3. Droplet Drying Method with and without Plasmon Resonance Hotspots on a Plasmonic Substrate

The study evaluated the droplet drying method using a plasmonic substrate both with and without laser assistance. A 5 mW He-Ne laser of 785 nm excitation wavelength was utilized to induce precipitation on the SERS substrate at room ambient conditions. To record the video of the precipitation process, a Leica SP8 confocal fluorescence microscope was used in fluorescence recovery after photobleaching (FRAP) mode. The following lasers were used: 405 nm laser at 5 mW, 488 nm laser at 2 mW, 552 nm laser at 2 mW, and 638 nm laser at 3 mW.

### 2.4. Raman Spectroscopy

The Raman mapping parameters were tailored to suit the specific requirements of this study:

- Sample Preparation: 5 μL of each sEV sample solution was deposited on the SERS substrate and dried before Raman testing.
- Spectrometer: Measurements were performed using a Reinshaw inVia Raman spectrometer at room temperature, with a laser excitation wavelength of 785 nm and a power of 5 mW.
- Calibration: The system was calibrated using the 520 cm^−1^ peak of silicon.
- Rough Mapping: Initial scouting for sEV locations was performed at a step width of 2 μm, with an exposure time of 0.2 s to prevent sample overheating.
- Fine Mapping: After spotting an sEV, fine mapping was performed at a step width of 0.1 μm to collect characteristic spectra from the sEV sample, maintaining the exposure time of 0.2 s to avoid overheating.

### 2.5. Polystyrene (PS) Beads

To simulate the dynamics of sEVs in fluid, PS beads were obtained in Milli-Q water from Alpha Nanotech Inc. The beads had a diameter of 100 nm, with a content of 10 mg/mL and a density of 1.05 g/mL. Based on the provided specifications, the bead concentration was calculated to be approximately 1.8 × 10^13^ particles/mL. Additionally, two types of Fluorescent Polystyrene Beads were acquired from SIGMA-ALDRICH: Alexa 488 and Alexa 647 conjugated. They have a diameter of 500 nm, a content of 2.5%, and a density of 1.05 g/mL. From the provided specifications, the initial bead concentration was determined to be about 3.6 × 10^11^ particles/mL. For the fluorescent imaging experiments, these beads were diluted 1000-fold using PBS, resulting in a final concentration of 3.6 × 10^8^ particles/mL.

### 2.6. Phosphate Buffered Saline (PBS)

PBS was sourced from SIGMA-ALDRICH. Detailed ingredients and concentrations are presented in Table S1 (Supplementary Materials).

### 2.7. Biological-Simulated Sample Solution

A simulated biological sample solution was prepared using PS in either PBS or deionized (DI) water. The detailed composition of this solution is available in Table 1. Considering that the concentration of exosomes in typical biological samples is approximately 10^8^ particles/mL, the chosen concentration for our simulated sample solution is deemed appropriate.

## 3. Results and Discussion

### 3.1. Challenges Limiting Throughput in SERS Analysis

As stated in the introduction, the foundational concern underlying the intricacies of SERS analysis for sEVs is the necessity for the colocalization of SERS hotspots with individual sEVs. In fact, the capability of SERS to detect and analyze at the single-vesicle level becomes meaningful only when sEVs align with the hotspot precisely, at the 100 nm scale [6,24]. Missing this critical alignment would be akin to searching for a needle in a haystack, thus largely reducing the potential throughput.

We now discuss the two most significant challenges contributing to limiting the potential of the SERS biosensing platform.

Challenge 1—Precipitation Challenge: The LSPR-induced electromagnetic-field enhancement enabling the SERS effect occurs in the proximity (few tens of nm) of the SERS substrate. Consequently, any build-up of material, such as the salt contained in the droplet, would locally “switch off” the Raman signal enhancement required for the detection of sEVs. Therefore, the precipitation phenomena occurring during the SERS analysis process can severely limit the throughput by covering a large portion of the SERS substrate.

**Figure 1:**
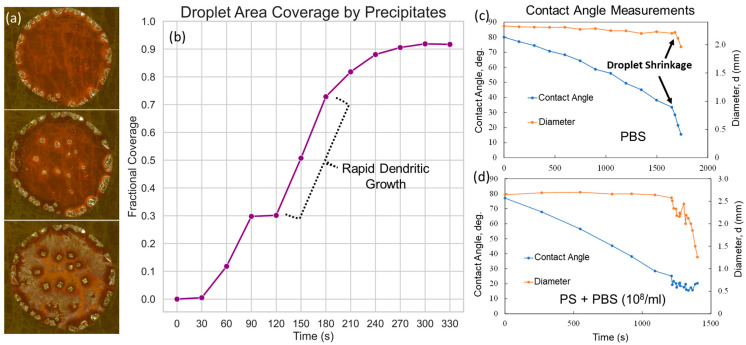
Crystallization dynamics on SERS substrate: (**a**) sequential progression of crystallization observed as the analyte droplet (PS in PBS) undergoes drying on a SERS substrate, illustrating the key stages of the process; (**b**) area covered by precipitates as the drying process progresses; (**c**,**d**) contact angle and droplet diameter.

This challenge manifests through the coffee ring effect and highlights the need to control precipitation spread. In the optical microscopy images of Figure 1a, we can observe the formation of dendritic structures during the droplet drying, covering a significant portion of the droplet footprint and, consequently, leading to a decreased surface available for SERS analysis, hence limiting the throughput. As calculated in Figure 1b, as soon as the drying process initiates, the salt precipitates rapidly cover the droplet area, with the majority of coverage from dendrites. The total coverage reaches over 90%. Optical microscopy images for each step have been provided in Figure S1 (Supplementary Materials).

Challenge 2—Technological Challenge: A second technological challenge revolves around the inherent trade-off between signal-to-noise ratio (SNR) and collection time per pixel in the Raman spectrometer setup. Following is the SNR vs. collection time trade-off: The Raman mapping process’s duration becomes a limiting factor when seeking to achieve a high SNR. A higher collection time per pixel is required to obtain better signal quality. However, this increase in collection time significantly limits the sample area that can be covered in a feasible timeframe. The SNR vs. collection time trade-off allows us to cover only approximately 1% of the sample area in the timeframe. This constraint underscores the need for technological advancements or alternative methods to reconcile the need for high-quality data with the requirement for higher throughput.

These challenges collectively contribute to the limitation in throughput and addressing them is vital for the successful deployment of SERS in biosensing applications. In the following sections of this paper, we will detail our innovative approach using lasers to induce precipitation and control these challenges, paving the way for enhanced throughput and reliability in SERS-based biosensing.

To overcome the identified challenges above, we present a novel approach involving localized laser-assisted plasmonic nucleation and the inherent purification mechanism in the growth process. This methodology aims to enhance throughput in SERS by addressing both the precipitation challenge and the technological challenge.

We present results on the utilization of lasers to reduce the nucleation barrier. The application of lasers in this context serves as a critical component of our methodology. The usage of a plasmonic substrate further aids in this process by enhancing the electromagnetic field at the surface. The synergy between the lasers and the plasmonic substrate effectively tailors the nucleation process.

The thermodynamics and kinetics underlying the growth process provide an intriguing aspect of our methodology. As crystallization occurs, foreign objects such as contaminants are often expelled from the growing crystal. This inherent purification mechanism can be harnessed for specific applications like exosome analysis. Our study delves into the thermodynamic principles and kinetic reactions involved, explaining how the growth process can be guided to expel foreign substances, thus serving as an inherent purification method.

### 3.2. SERS Spatial-Overlap Study

To elucidate the purification mechanism manifested in the dynamics of drying and the resultant variations in contact angle analysis across different materials, we first turn our attention to the observed phenomena in the coffee ring effect, a crucial aspect vividly captured in Figure 1. As shown in the contact angle measurements, we see that the droplet diameter remains fixed up to a point; however, the contact angle keeps decreasing monotonically. This demonstrates the pinning effect, which is an identifying characteristic of the coffee ring effect [25,26]. In fact, we have observed coffee ring formation for different types of solutions, suspensions, and colloids as presented in Figure 1a for PS in PBS, Figure S2a,b (Supplementary Materials) for PBS and PS in deionized (DI) water, and subsequent sections for sEVs in PBS.

**Figure 2:**
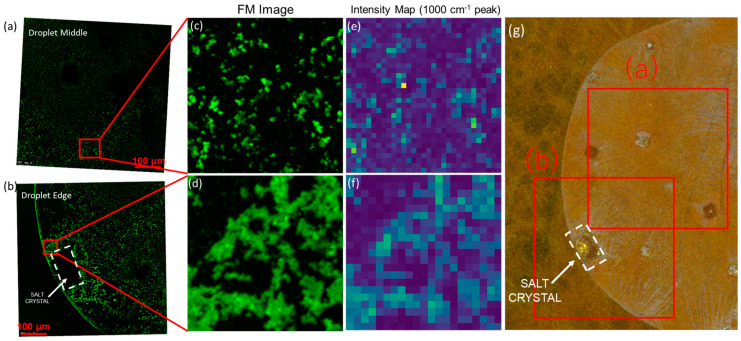
Confocal FM image of Alexa 488-conjugated polystyrene beads forming a coffee ring (**a**,**c**) at the droplet center and (**b**,**d**) at the droplet edge. (**e**,**f**) At the corresponding locations: SERS mapping of the 1000 cm^−1^ peak of polystyrene which is linked to the breathing mode of the aromatic carbon ring of styrene; (**g**) optical microscopy images with the locations presented in (**a**,**b**) marked in red.

Following the delineation of the drying dynamics, we aim to shed light on the rationale behind the PS bead analysis undertaken in this study. To this end, SERS spatial-overlap studies were conducted to further validate that the bright spots observed are indeed the beads. Since PS has been used in past studies to simulate the dynamics of sEVs in biosamples [27,28,29,30], we expect a similar behavior of sEVs as observed for PS beads in our studies. As illustrated in Figure 2, the SERS spatial-overlap studies were embarked upon to ascertain the nature of the bright spots observed, hypothesized to be the beads. A concerted effort was made to correlate the spatial locations of these PS beads with the SERS signals of PS [31], utilizing fluorescence microscopy to establish a clear linkage between the observed bright spots and the beads themselves. This correlation, clarified further in the forthcoming sections, serves as a potent scientific foundation, bridging the existing gap between the structural insights rendered by SERS and the spatial information gleaned from fluorescence microscopy, thereby painting a comprehensive picture of the purification mechanism in play. This correlation not only confirms the nature of the observed bright spots but also offers a robust scientific basis for understanding the interplay between crystal growth and molecular displacement. Another key observation is of the higher density of PS beads near the droplet edge (Figure 2d), as compared to the center (Figure 2a), which is further validation of the coffee ring formation process where the outward convective flow of particles due to capillary action is a key mechanism.

Furthermore, as delineated in Figure 2d, there is a discernible absence of colocalization between the PS beads and salt crystals. This observation serves as a critical foundation for the subsequent sections where we intend to exploit this distinct segregation behavior to facilitate the targeted accumulation of sEVs within a confined region at the periphery of the plasmonic-induced precipitates.

### 3.3. Controlled Nucleation: Plasmonic-Induced Precipitation on SERS Substrate

The controlled nucleation and localized precipitation of salt on the SERS substrate is a focal point of this research. By strategically exciting the location of plasmonic hotspots on the substrate surface, we were able to achieve a groundbreaking result. Below, we present the insights drawn from our results and discuss the underlying processes.

#### 3.3.1. Coffee Ring Formation Mechanism

As shown in Figure 1 and Figure 2, we observe the coffee ring mechanism as the analyte droplet dries up on our substrate. In Figure 1c,d, we present the contact angle analysis for different samples, revealing the presence of the coffee ring effect in the samples, characterized by the formation of dendritic structures that consume a significant part of the surface area (over 90%, as calculated in Figure 1b). The pinning of the three-phase contact line at the molecular level lies at the heart of this effect. During the drying process, the liquid’s perimeter becomes anchored to specific nucleation sites or defects on the substrate. These pinning sites are often the result of molecular interactions between the liquid and solid surface, coupled with the particular geometric or chemical heterogeneity of the surface. This molecular pinning maintains the droplet diameter and leads to the formation of dendritic structures that maximize the covered area. By restricting the liquid perimeter’s movement, the pinning effect accentuates the deposition of solutes at the edges, giving rise to the coffee ring effect [25,26,32,33,34].

Toward the end of the drying process, as the chemical potential of the solute in solution form diminishes in the pinned region due to precipitation, the molecular forces responsible for pinning become less dominant. As a result, the reduced contact angle contributes to an increase in the projected force along the water–substrate interfacial plane. This increased force, akin to an increased solid–liquid interfacial tension, leads to the contact line’s recession. The rationale here is that a reduction in the contact angle effectively increases the component of the force along the interfacial plane of the water–substrate surface energy, breaking the force balance at the three-phase line, as demonstrated in Figure S3 (Supplementary Materials). Furthermore, at the contact line, where the liquid meets the solid surface, surface tension plays a dominant role. It acts tangentially to the liquid–air interface and pulls the liquid inward. This force works to minimize the surface area of the liquid. Hence, as the contact angle reduces due to drying, the inward force from the surface tension overcomes the pinning force, and the contact line starts to recede. The droplet’s diameter begins to shrink, and the liquid retracts toward the center of the droplet. This molecular-level understanding provides a basis for the observed diameter reduction, a critical insight for optimizing the surface area availability for SERS analysis.

#### 3.3.2. Plasmonic-Induced Precipitation

The key here lies in the unique properties of plasmonic materials, which allow for a resonant interaction between the laser light and the surface plasmons [35]. The localized surface plasmon resonance (LSPR) leads to an enhancement in the electromagnetic field at the surface, which drives the nucleation process [36,37].

**Figure 3:**
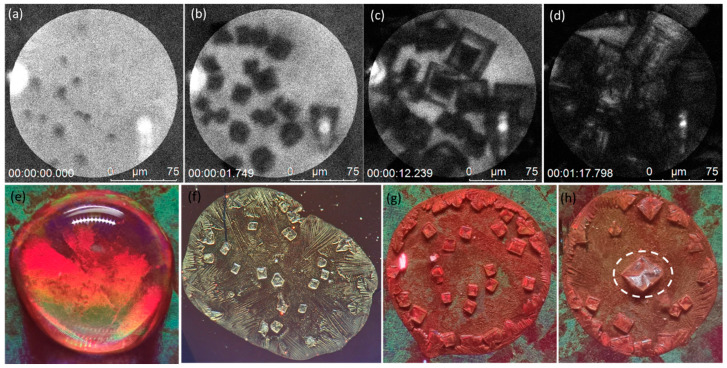
(**a**–**d**) Progress of nucleation and growth observed on the SERS substrate at the laser spot, (**e**) analyte droplet containing fluorescent PS beads, (**f**) no localized precipitation observed on the flat-gold substrate which is non-plasmonic, (**g**) dried droplet on SERS substrate without the laser, (**h**) dried droplet on SERS substrate with the laser.

This localized electromagnetic-field intensification facilitates the lowering of the nucleation barrier, driving a more controlled precipitation at the specific locations. It also highlights the inherent properties of plasmonic materials that amplify localized fields, a characteristic absent in a non-plasmonic substrate such as unstructured Au or Si substrates, as shown in Figure 3f and Figure S4 (Supplementary Materials). The mechanism behind localized nucleation through laser-induced plasmonic processes is inherently complex. It involves an interplay between the laser parameters, plasmonic substrate properties, and the solute’s physical chemistry.

1. Laser Contribution on Plasmonic Substrate:

Lasers provide a directed energy source. When a laser interacts with a substrate, it creates an electromagnetic field that can cause local heating. The localized increase in temperature enhances the kinetic energy of the molecules in the vicinity. By increasing the temperature in a specific region, localized heating does indeed increase the kinetic energy and entropy of the molecules. This higher entropy would typically oppose the formation of ordered structures like a stable nucleus. However, nucleation is a balance between entropy and enthalpy. We expect that the enthalpy gain from forming the new phase is sufficiently high, and it outweighs the entropy term, leading to a net decrease in the free energy barrier for nucleation [38]. Although, it must be stated the understanding of this phenomenon is currently incomplete and deserves further study. Having said that, our results are further corroborated by the repeated observation of laser-assisted nucleation experimentally, as shown in Figure S5 in the Supplementary Materials. Furthermore, the localized heating could provide enough kinetic energy for the molecules to overcome short-range repulsive interactions and allow them to reach the configuration where attractive forces dominate. This is especially pertinent as the system reaches supersaturation due to the continuous evaporation. The energy barrier equation is obtained from classical nucleation theory (CNT) [39]. According to CNT, the Gibbs free energy change ∆G for the formation of a nucleus of radius *r* is given by

(1) $$∆G=4\pi r^{2}\gamma -\frac{4\pi r^{3}{∆G}_{v}}{3}$$

where γ is the surface energy per unit area, *r* is the nucleus size, and ${\Delta G}_{v}$ is the change in Gibbs free energy per unit volume for the transformation. By calculating for r at the minimum gradient of ΔG, we obtain the nucleation barrier ${\Delta G}^{*}:$

(2) $${\Delta G}^{*}=\frac{16\pi \gamma^{3}}{3{{\Delta G}_{v}}^{2}}\;$$

Local heating effectively reduces γ, thereby reducing the nucleation barrier. To elaborate, γ is reduced because at elevated temperatures the increased kinetic energy of molecules leads to more significant molecular vibrations. These can disrupt the regular lattice structure of a crystalline nucleus. The disruption in structure leads to more dangling bonds, which causes a higher surface energy density. However, what reduces the surface energy density at high temperature is the larger average distance of dangling bonds due to increased vibration and thermal expansion [40]. Furthermore, it must be noted that the plasmonic contribution is essential for the localized nucleation, as evidenced in Figure 3.

Another point to be addressed here is that heating should also increase the solubility, or alternatively, increase the randomness, i.e., favoring a higher entropy state, which is the opposite of crystal formation, which is true as generally for many solutes in a solvent, as temperature increases, solubility also increases due to the endothermic nature of the dissolution process. This implies that the dissolved molecules prefer the increased kinetic energy that comes with higher temperatures. Yet, the immediate and intense heating around a plasmonic nanostructure leads to a local reduction in the chemical potential of the solute species [41], which creates a strong driving force for the internal diffusion of the solute molecule. This could momentarily drive the local concentration of the solute beyond its solubility limit, creating a supersaturated condition, and hence, localized nucleation.

However, the energy solely from the laser, without any form of localized enhancement, is not sufficient to locally reduce the kinetic barriers for nucleation. Hence, we utilize our plasmonic nanopyramid substrate to this end.

2. LSPR Contribution:

Plasmonic substrates amplify the laser’s effect through the phenomenon of localized surface plasmon resonance (LSPR). LSPR is a resonant oscillation of conduction electrons at the interface between negative and positive permittivity material stimulated by incident light. LSPR effects induce local electromagnetic-field enhancements. Specifically, the enhancement factor for the electric field, often denoted by $F_{E}$, is typically in the order of 10–100. However, when we refer to the SERS enhancement of the Raman signal, which scales as $E^{4}$, the SERS enhancement factor can lie in the range of $10^{4}$–$10^{8}$ [4,42]. At the substrate surface, the incident beam intensity is greatly enhanced by the following relation:

(3) $$E_{localized}=E_{incident}\times F_{E}\;$$

where $E_{localized}$ is the localized electric-field intensity, and $E_{incident}$ is the incident electric-field intensity. When considering Joule heating due to the local electric field, the enhancement of the dissipated power scales as $E^{2}$, suggesting enhancement levels up to $10^{4}$. The temperature increases according to the heat equation, considering both the absorbed laser energy and the specific heat properties of the materials:

(4) $$\rho C_{p}\frac{dT}{dt}=\nabla .\left(K\nabla T\right)+\sigma {\left|E_{localized}\right|}^{2}$$

where ρ is the density of the material, $C_{p}$ is the specific heat capacity at constant pressure, K is the thermal conductivity, σ is the absorption cross-section for the gold nanopyramids, and T is the temperature.

The combination of the laser energy with the plasmonic substrate is instrumental in making localized nucleation easier. The enhanced electric field near the plasmonic substrate due to LSPR leads to a higher gradient in concentration and temperature, thereby increasing the nucleation rate [41], as given by the Arrhenius relation:

(5) $$N=N_{0}e^{-\frac{{\Delta G}^{*}}{kT}}\;$$

where N is the nucleation rate, $N_{0}$ is the pre-exponential factor, *k* is the Boltzmann constant, and *T* is the temperature. The localized heating reduces ${\Delta G}^{*}$ and increases T, thereby increasing the nucleation rate specifically at that location.

While our experiments consistently provided the counterintuitive result of localized precipitation, upon laser interaction, thermodynamic reasoning suggested a different outcome. One would typically anticipate, based on entropy-driven arguments, that the increased temperatures would promote dissolution, opposing localized nucleation. Indeed, in most scenarios, higher entropy favors dissolution over the creation of ordered structures. Yet, the experimental observation is the unvarnished truth.

Our hypothesis revolves around a kinetics-based argument. While thermodynamics tell us the favorable state of a system, kinetics dictate how we get there. One plausible explanation lies in the driving force initiated by a localized decreased chemical potential, ushering in an inward flux of solute [41,43]. This localized migration, under certain conditions, could lead to a supersaturation state. In our experiments, the intense localized heating around the plasmonic nanostructure, brought about by the confluence of laser energy and surface plasmon resonance (SPR), seems to momentarily diminish the solute species chemical potential. This sudden drop instigates a strong internal diffusion of the solute molecules. The rapid influx can momentarily elevate the local concentration of the solute beyond its thermodynamic solubility threshold. An added factor is the possible formation of nanobubbles induced by high-energy plasmonic hotspots [44,45,46]. These act as heterogeneous nucleation sites which allow for much lower nucleation barriers. Hence, we believe such transient supersaturated conditions become hotbeds for localized nucleation. The growth here occurs only within the first few nm from the SERS substrate where the heat is; hence, the material builds up from the bottom to the top, driven by the inward diffusion of solute due to the chemical potential gradient.

To conclude, while the broader thermodynamic landscape might suggest dissolution as the pathway of least resistance, the kinetics, steered by our experimental conditions, defies this by fostering conditions ripe for localized precipitation. The powerful synergy between electromagnetic fields and SPR, achievable uniquely through our experimental setup, has unveiled a novel nucleation-promoting mechanism.

Through the precise control and understanding of these processes, we can harness this complex phenomenon for enhancing biosensing applications. The strategy of targeted nucleation helps in minimizing unwanted precipitate coverage and maximizing the signal’s quality and quantity, setting a new standard in the field of biosensing. Hence, we illustrate how the unique optical and thermal properties of the nanopyramid gold substrate might reduce the nucleation barrier through plasmonic effects.

### 3.4. Segregation Mechanism of the Growth Process

**Figure 4:**
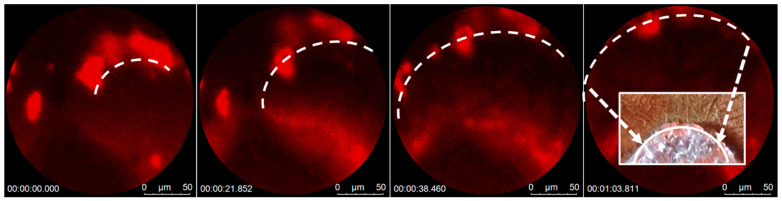
Cluster of Alexa 647-conjugated PS beads being pushed away by crystal nucleation and growth at the laser spot on the SERS substrate. The dashed line indicates the perimeter of a crystallite, which moves with the growth process. A True color image of the precipitate is inset on the right. A reduced focal length at higher magnification leads to variable focusing as the droplet height reduces as it dries.

As salt crystals form on a substrate, they tend to bond more strongly with each other compared to other molecules present in the solution. This preference leads to a natural purification mechanism, where unwanted molecules, such as exosomes or other vesicles, are “pushed out” of the crystallization region. As shown in Figure 4, this effect has been observed and recorded using advanced microscopy techniques, including fluorescence microscopy (FM) with the fluorescence recovery after photobleaching (FRAP) setting of a Leica Confocal SP8 fluorescence microscope.

FM images of fluorescent polystyrene (PS) beads were used to directly visualize this purification process. These images reveal the formation of salt crystals and the accompanying displacement of PS beads. As the salt molecules nucleate and grow, they form specific crystal lattice structures, and their mutual attraction becomes significant. The FM images provide time-resolved insights into this phenomenon, showing the step-by-step process through which the beads are displaced by the forming salt crystals.

The purification mechanism can be understood through the principles of intermolecular forces and crystal growth. As salt crystals form, the strong ionic bonds between the salt molecules dominate the weaker van der Waals or other secondary interactions with the PS beads. As the salt crystals grow, they continue to favor bonding with other salt molecules, leading to a physical displacement of the PS beads, akin to a “pushing-out mechanism”. This purification mechanism observed during the salt crystallization process provides a striking example of the interplay between crystallography, intermolecular forces, and material properties, revealing how crystal growth can lead to natural purification.

In the realm of biosensing, employing this process could significantly enhance signal throughput and provide a more precise determination of bioparticle locations. These bioparticles are likely to cluster around the peripheries of the induced precipitates with high areal density, a phenomenon driven by the aforementioned mechanisms.

These findings not only deepen our understanding of crystallization and purification processes but also hint at potential applications in areas such as selective separation, biosensing, and materials engineering. By harnessing this inherent purification mechanism, novel methods may be developed to isolate specific molecules or particles within complex mixtures, paving the way for new technological advancements in various scientific and industrial domains.

### 3.5. Reduced Crystallization Area Coverage and Increased Throughput

**Figure 5:**
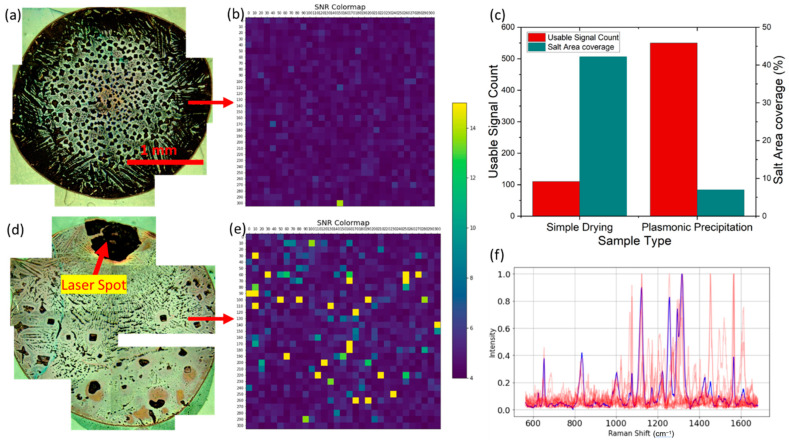
Analysis of an analyte droplet containing sEVs: (**a**) composite image of a simply dried droplet, (**b**) areal mapping showing the low density of good SNR spectra; (**c**) 5× rise in the number of usable SERS signals after laser-assisted drying; (**d**) laser-assisted drying; (**e**) higher areal density of high SNR peaks observed, and there is an inverse relation to salt area coverage, which is reduced due to plasmonic precipitation; (**f**) overlap of 10 high-quality SERS signals observed from sample (**d**).

In Figure 5, we demonstrate a significant reduction in crystallization area coverage due to localized precipitation, thus enhancing the throughput. This can be attributed to the conservation of the mass of solute.

If precipitation is concentrated in specific locations, less solute will be deposited in other areas. This is advantageous for biosensing applications because fewer nanopyramids will be covered by the precipitation, allowing more exosomes to interact with hotspots. The net result is an increase in the quality and quantity of signals captured.

The relationship can be understood through the equation for mass conservation, at a given instant:

(6) $$\rho_{solute}\times V_{solute}=constant\;$$

where $\rho_{solute}\;$is the density of the solute, and $V_{localized}$ is the volume in which precipitation is localized. Due to diffusion toward the laser spot, the solute concentration elsewhere in the bulk reduces, leaving less solute to cover up the substrate through precipitation, as shown in Figure 5d.

By controlling the volume through the application of localized laser energy on a plasmonic substrate, we ensured that the solute was concentrated at specific locations, reducing the spread. This unprecedented control of nucleation enabled us to maintain the same mass of solute but reduce the surface area coverage, leaving more nanopyramids exposed for interaction with exosomes.

The observed controlled localized precipitation on a plasmonic substrate arises from the synergistic effects of laser-induced localized heating and the plasmonic properties of the nanostructured gold substrate. This process’s complexity necessitates a careful balance between various factors, each contributing to the reduction in the nucleation barrier. The successful control of this plasmonic-induced nucleation process leads to a reduction in crystallization area coverage and an increase in throughput of five times. By ensuring that less of the nanopyramids are covered by precipitation, more sEVs will interact with hotspots, resulting in better and more signals.

Understanding and exploiting this delicate balance sets a new standard in the field of biosensing, illustrating the potent combination of physics, chemistry, math, and materials science to unravel and control complex natural phenomena.

## 4. Summary and Conclusions

In this research, a pioneering approach to biosensing has been developed by coupling localized nucleation induced by lasers on a plasmonic substrate with a novel purification mechanism. The intricacies of the plasmonic effects allowed for precise control over localized precipitation. This holds the potential to significantly enhance the throughput of any sensing application utilizing the droplet drying method. Specifically, the two reasons for higher throughput are (a) the greater availability of substrate for the colocalization of hotspots with biomarkers and (b) the clustering of particles around these precipitates with high areal density, providing high signal density at these locations. The principles and methods elucidated in this work provided a pathway for controlled nucleation via the LSPR-assisted selective reduction in nucleation barriers. An innovative purification mechanism, revealed through fluorescence microscopy and SERS spatial-overlap studies, led to more robust and higher throughput signals for biosensing. The implications of this research extend to groundbreaking advancements in disease diagnosis and environmental sensing, emphasizing the importance of interdisciplinary innovation in materials science and optics. The complex underlying physics and chemistry offer promising avenues for further exploration and application. Overall, this work represents a substantial advancement in biosensing methodologies, especially the various sensing applications that rely on droplet drying.

## Figures and Tables

**Table 1:** Simulated solutions used in Figure 1.

PS Concentration, Particles/mL	Solution
$1.8\cdot 10^{13}$	DI
$1.8\cdot 10^{11}$	DI or PBS
$1.8\cdot 10^{8}$	DI or PBS
0	DI or PBS

## Data Availability

Data are contained within the article.

## Supplementary Materials

The following supporting information can be downloaded at https://www.mdpi.com/article/10.3390/bios13121016/s1, Figure S1. Progression of crystallization; Figure S2. Coffee-ring formation for (a) PBS solution, (b) PS in DI water; Figure S3. Force balance for surface tension components at the contact line at droplet-substrate interface; Figure S4. No laser-induced precipitation observed on (a) Unpatterned Au substrate, (b) Silicon substrate; Figure S5. Repeated observation of plasmonic precipitation; Table S1. PBS Composition.
