# Supplementary material for: Controlling the Nucleation and Growth of Salt from Bodily Fluid for Enhanced Biosensing Applications
Source: Biosensors (Basel). 2023 Dec 6;13(12):1016. doi: 10.3390/bios13121016 (PMC10741434; doi:10.3390/bios13121016)
Supplement: Supplementary file 1 [file biosensors-13-01016-s001.zip › biosensors-2750691-supplementary.pdf]

Controlling the nucleation and growth of salt from bodily fluid for enhanced biosensing application

Siddharth Srivastava [1], Yusuke Terai [1,2], Jun Liu [1], Giovanni Capellini [3,4] and Ya-Hong Xie [1,5,*]

[1] Department of Materials Science and Engineering, University of California, Los Angeles, CA 90095, USA; sidsri@ucla.edu (S.S.); yterai1105@gmail.com (Y.T.)
[2] Department of Micro-Nano Mechanical Science and Engineering, Nagoya University, Nagoya 464-8601, Japan
[3] IHP—Leibniz Institute for High Performance Microelectronics, 15236 Frankfurt (Oder), Germany; giovanni.capellini@uniroma3.it
[4] Department of Science, Università Degli Studi Roma Tre, Viale Marconi, 446 Rome, Italy
[5] Jonsson Comprehensive Cancer Center, University of California, Los Angeles, CA 90095, USA
* Correspondence: yhx@ucla.edu

SUPPLEMENTARY DATA

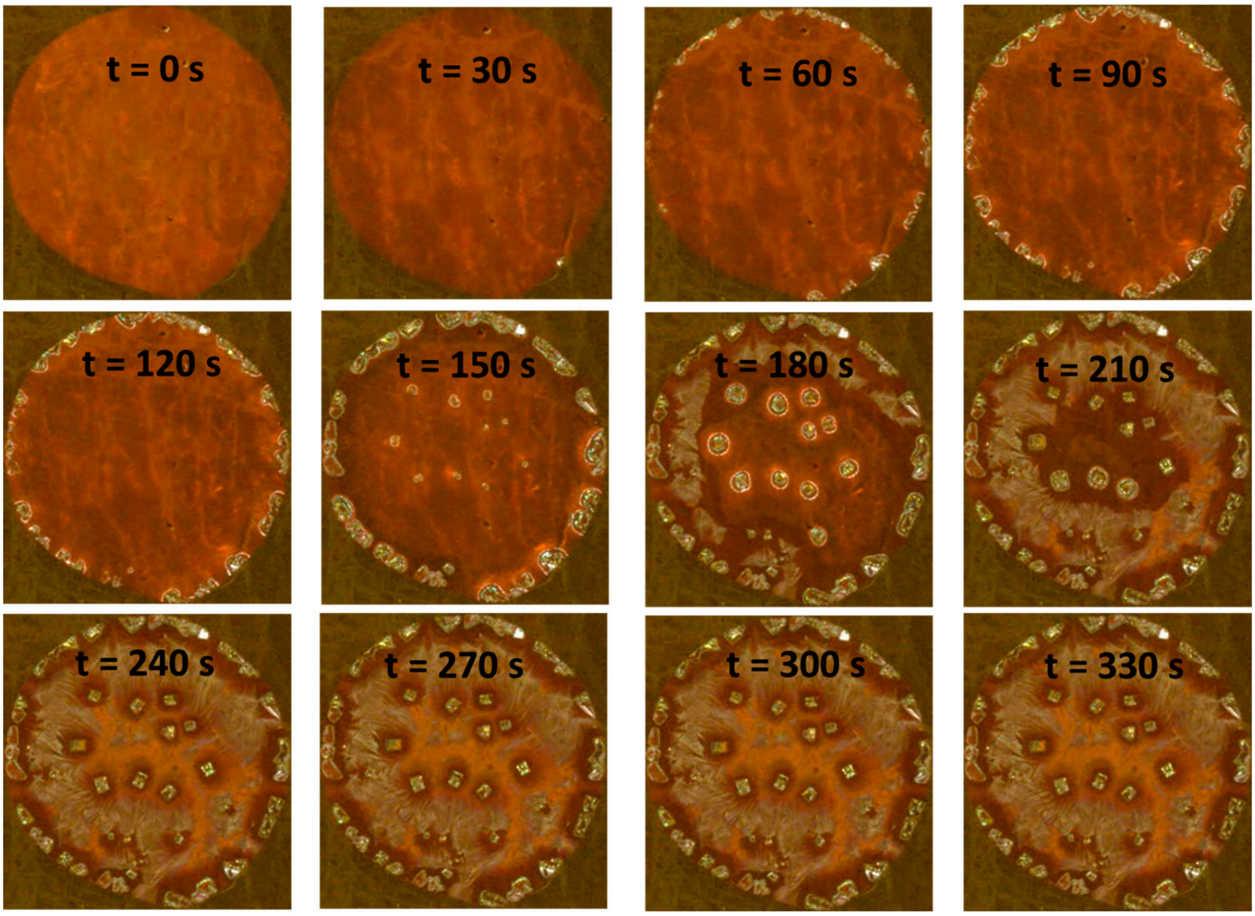

Figure S1. Progression of crystallization

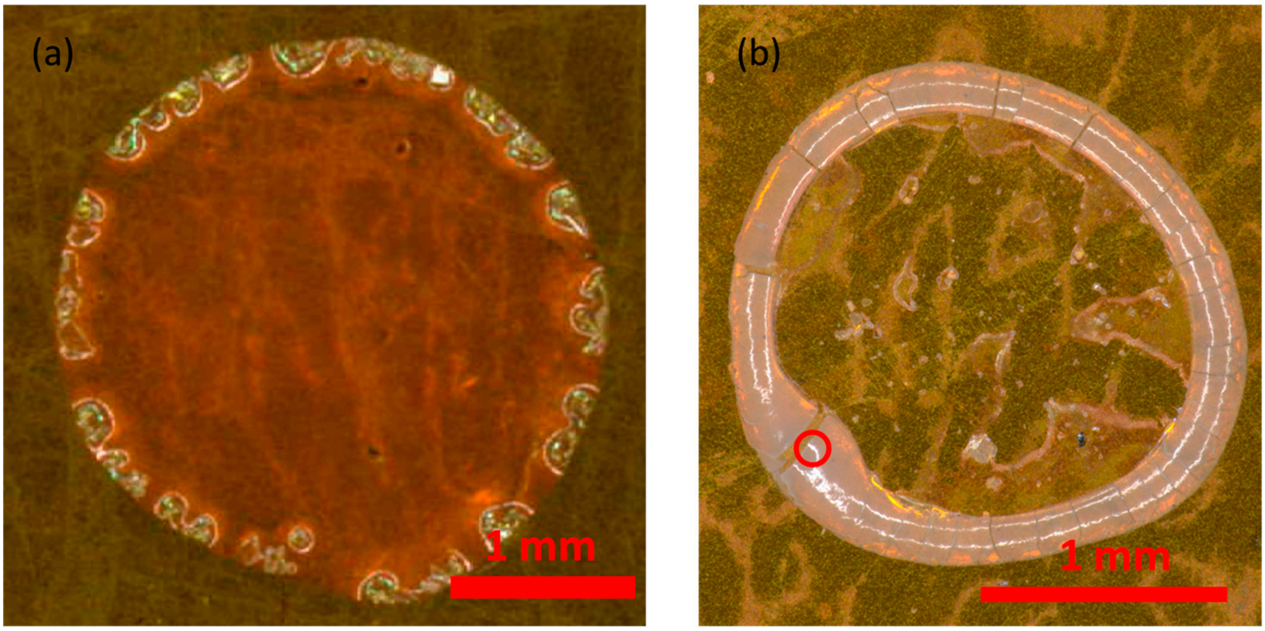

Figure S2. Coffee-ring formation for (a) PBS solution, (b) PS in DI water

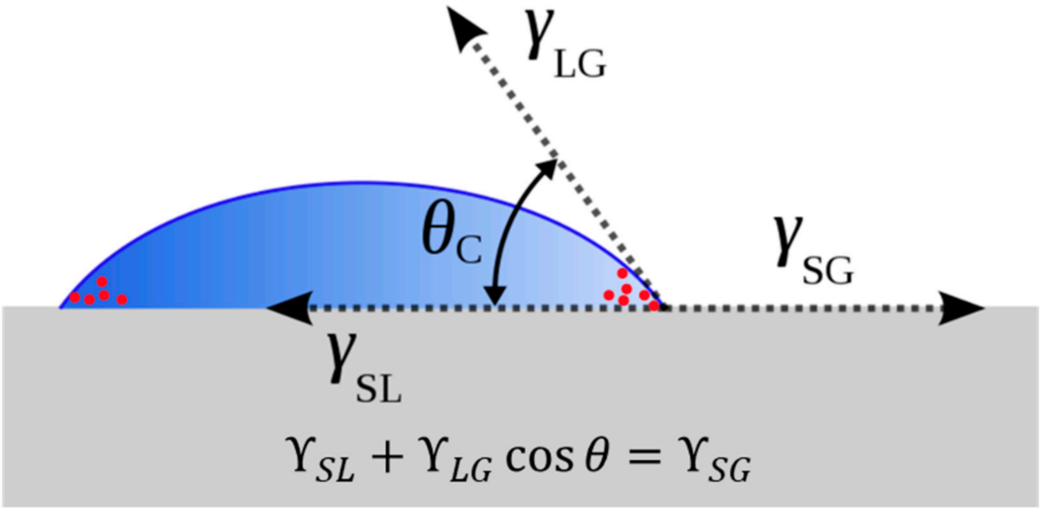

Figure S3. Force balance for surface tension components at the contact line at droplet-substrate interface

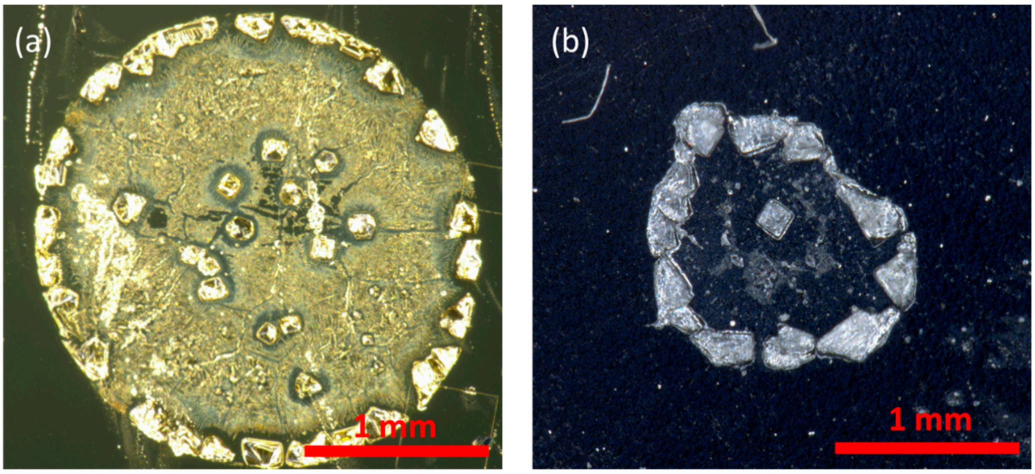

Figure S4. No laser-induced precipitation observed on (a) Unpatterned Au substrate, (b) Silicon substrate

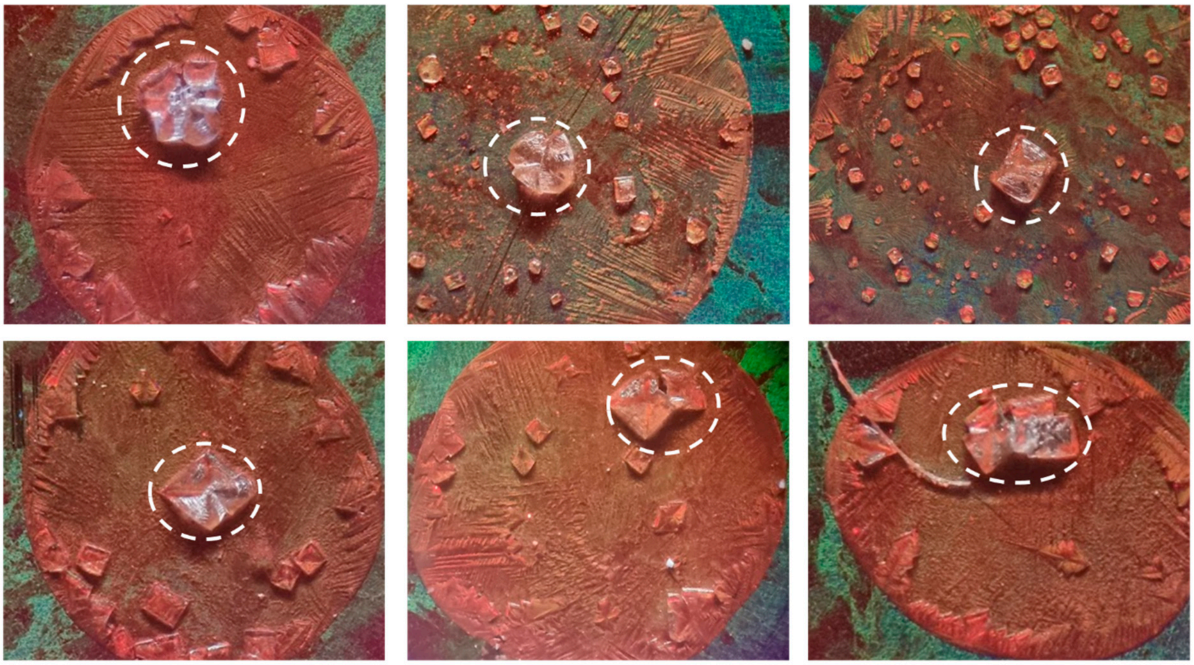

Figure S5. Repeated observation of plasmonic precipitation

Table S1. PBS Composition

Reagent	Amount, g	Concentration, mM
NaCl	8	137
KCl	0.2	2.7
Na_2HPO_4	1.44	10
KH_2HPO_4	0.24	1.8
